# Genetic relatedness of faecal coliforms and enterococci bacteria isolated from water and sediments of the Apies River, Gauteng, South Africa

**DOI:** 10.1186/s13568-016-0319-4

**Published:** 2017-01-07

**Authors:** Mutshiene Deogratias Ekwanzala, Akebe Luther King Abia, Eunice Ubomba-Jaswa, Jitendra Keshri, Ndombo Benteke Maggy Momba

**Affiliations:** 1 Department of Environmental, Water and Earth Sciences, Tshwane University of Technology, Arcadia Campus, Private BagX680, Pretoria, 0001 South Africa; 2 Natural Resources and the Environment, CSIR, PO Box 395, Pretoria, 0001 South Africa

**Keywords:** *Enterococcus* spp, Faecal coliforms, Genetic similarity, River water, Riverbed sediment

## Abstract

To date, the microbiological quality of river sediments and its impact on water resources are not included in the water quality monitoring assessment. Therefore, the aim of this study was to establish genetic relatedness between faecal coliforms and enterococci isolated from the river water and riverbed sediments of Apies River to better understand the genetic similarity of microorganisms between the sediment and water phases. Indicator bacteria were subjected to a molecular study, which consisted of PCR amplification and sequence analysis of the 16S rRNA and 23S rRNA gene using specific primers for faecal coliforms and enterococci, respectively. Results revealed that the Apies River had high faecal pollution levels with enterococci showing low to moderate correlation coefficient (r^2^ values ranged from 0.2605 to 0.7499) compared to the faecal coliforms which showed zero to low correlation (r^2^ values ranged from 0.0027 to 0.1407) indicating that enterococci may be better indicator than faecal coliforms for detecting faecal contamination in riverbed sediments. The phylogenetic tree of faecal coliforms revealed a 98% homology among their nucleotide sequences confirming the close genetic relatedness between river water and riverbed sediment isolates. The phylogenetic tree of the enterococci showed that *Enterococcus faecalis* and *Enterococcus faecium* are the predominant species found in both river water and riverbed sediments with bootstrap values of ≥99%. A high degree of genetic relatedness between sediment and water isolates indicated a possible common ancestry and transmission pathway. We recommend the microbial monitoring of riverbed sediments as it harbours more diverse microbial community and once resuspended may cause health and environmental problems.

**Electronic supplementary material:**

The online version of this article (doi:10.1186/s13568-016-0319-4) contains supplementary material, which is available to authorized users.

## Introduction

The South African government has implemented regulations and policies to deliver safe water to all; however, based on the figures given in the Millennium Development Goals (MDG) Report 2013, 3–5 million people in South Africa still lack access to an improved water source (UN 2013). Many families in southern Africa including South Africa and especially those residing in non-metropolitan areas still rely on river water for drinking and other domestic purposes (Donovan et al. 2008; Abhirosh et al. 2010). Microbiological contamination of drinking water is one of the main causes in the spread of waterborne diseases (Franz 2005). Monitoring of riverbed sediments worldwide typically focuses on the analysis of the levels of chemical contaminants (Guerra et al. 2009), whereas the risks posed by the presence of pathogenic microorganisms within the sediment compartment are largely disregarded (Luna et al. 2010). Sediment re-suspension into the water column may cause debilitating waterborne diseases in individuals, particularly immunocompromised individuals (Elliot and Colwell 1985).

The degree of risk associated with waterborne pathogens is certainly high with the knowledge that pathogens can grow within the sediment compartments of aquatic systems (Jamieson et al. 2004). This risk can be exacerbated by current microbial monitoring policies which do not consider the phases of pathogens associated with aquatic sediments. Furthermore, there is a general lack of understanding of the nature and relevance of pathogen and sediment association and the risks of infection due to exposure of people to the pathogens mobilised from sediments, as well as pathogen viability, transport, and fate within aquatic systems (Droppo et al. 2009). Bacteria often show an affinity for sediment attachment (Liss et al. 1996; Jamieson et al. 2004) as sediments represent a beneficial environment for nutrient, food assimilation and protection from environmental stress such as contaminants and predation. Increasing evidence indicates that many marine sediments (stream substrates) serve as reservoir of pathogenic microorganisms of faecal origin (e.g. *Escherichia* and *Enterococcus*), including pathogenic and virulent strains of bacteria (Alm et al. 2003; Luna et al. 2010). The presence of these faecal bacteria poses serious concerns for the quality of aquatic systems as well as for human health, especially when sediments undergo re-suspension due to both natural and anthropogenic disturbances (Luna et al. 2012).

Several methods for studying genetic similarity have been developed throughout the years. Subsequently, some housekeeping genes such as the highly conserved 16S rRNA and 23S rRNA genes have been used. In spite of the fact that the outright rate of progress in the 16S rRNA gene evolution is not known, it marks evolutionary distance and similarity of organisms (Thorne et al. 1998). Others have found the utilisation of 23S rRNA groupings supportive in recognising similarity among *Streptococcus* spp. (Rantakokko-Jalava et al. 2000). Although few scientists agree on sole use of 16S rRNA gene sequences (Roth et al. 1998), others view the combination of 16S and 23S rRNA gene grouping as a great tool for phylogenetic analysis (Song et al. 2004).

Genetic similarity has been used widely in molecular microbiology and heredity studies in order to confirm molecular information gathered on pathogenic strains to establish their relationship with other bacteria isolated from different sources, such as that between clinical specimens and environmental isolates (Shayegani et al. 1991; Rood et al. 2011; Salama et al. 2012; Njage and Buys 2015). To date, the phylogenetic relationship has been used to reveal similarity among microbial isolates from many targets: food (van Megen et al. 2009); soil (Pester et al. 2012) and wastewater (Steinberg and Regan 2008), but as yet little is known about the genetic similarity between indicator bacteria of aquatic systems and those of riverbed sediments.

The aim of the present study was to establish the genetic similarity between faecal coliforms and enterococci isolated from river water and riverbed sediments of the Apies River.

## Materials and methods

### Study area

**Fig. 1:**
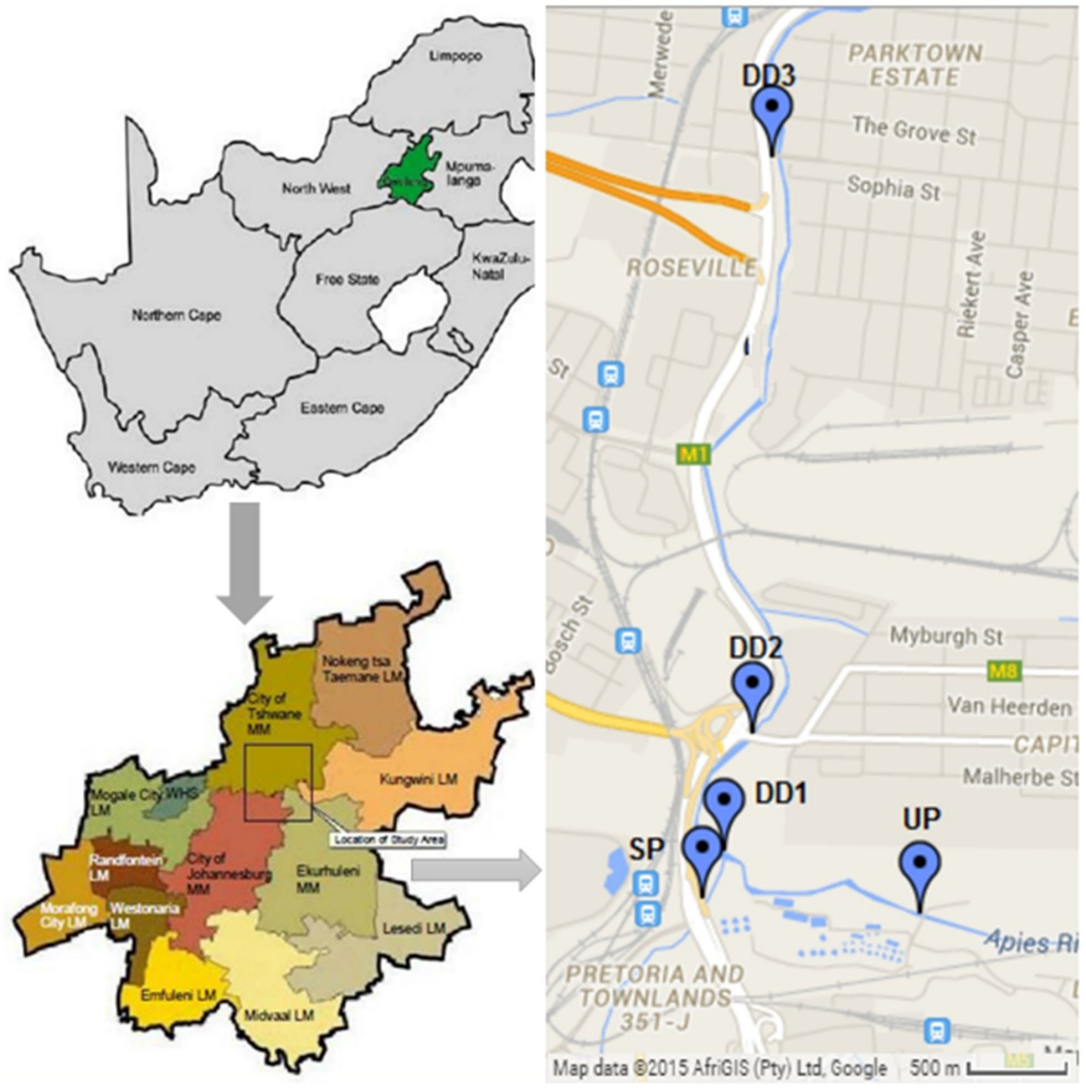
Sites location. Location of sampling sites along Apies River. *UP* upstream Daspoort Wastewater treatment plant (WWTP), *SP* skinner spruit, *DD1* downstream Daspoort Site 1, *DD2* downstream Daspoort Site 2, *DD3* downstream Daspoort Site 3

The Apies River is located in Pretoria in the Gauteng Province of South Africa and it is one of Tshwane’s significant natural resources. The river rises in the Fountains Valley, Pretoria and flows through Gauteng, North–West and Limpopo Provinces to ultimately join the Limpopo River. It falls within the Crocodile (West) Marico Water Management Area and has a total flow >500 m^3^ per year, which is controlled by diverse processes such as the treated effluents from four wastewater treatment works, the extraction of water for different usages and the total rainfall and runoff reaching the river. These wastewater treatment works discharge their treated effluents to the river and contribute roughly 12% of the stream flows. A number of land-use activities along the river starting from Pretoria Central, Arcelor-Mittal Steelworks in Pretoria-West and substantial parts of Atteridgeville contribute to the lower microbiological quality of this river (RHP RHP (River Health Programme) 2005; Abia et al. 2015c). Figure 1 illustrates various sites for the collection of water and sediment samples during the study. UP (upstream Daspoort Wastewater treatment plant-WWTP); SP: Skinner Spruit, effluent from Atteridgeville; DD1: downstream Daspoort Site 1, mixing point between Apies River and Skinner Spruit; DD2: downstream Daspoort Site 2, 1 km from the Daspoort WWTP; DD3: downstream Daspoort Site 3, 3 km from the Daspoort WWTP).

### Collection of water and sediment samples

A total of five sampling sites were selected along the river as illustrated in Fig. 1. The sampling programme was performed on a weekly basis from August to November 2014. Water samples were collected using sterile plastic containers, by wading out to a depth of 0.1 m, midway between the banks following standard procedures (US EPA 2002a, b). Grab sediment samples were collected from the top 5 cm of the riverbed at each sampling site, using a sterile polypropylene scoop and transferred to sterile 100 mL polypropylene containers with lids. All the samples were transported to the laboratory at 4 °C in cooler-boxes containing ice and analysed within 3 h of collection.

### Enumeration of culturable faecal coliforms and Enterococcus species

The membrane filtration technique was utilised for the cultivation and enumeration of faecal coliforms and *Enterococcus* spp. for both river water and riverbed sediments, following procedures described by Luna et al. (2010). The Chromocult® Coliform agar (Merck) and Chromocult® Enterococci agar (Merck) were used, respectively. In this study, the water displacement method previously described by Abia et al. (2015a) was used to quantify the target bacteria in the sediments, instead of the common approach (weighting method). Briefly; this approach which is based on Archimedes’ principle consist of gradually transferring sediment samples were into a graduated 1 L Durham bottle containing 900 mL of 1× PBS until the 1000 mL mark was reached to obtained a 10% dilution (v/v). Thereafter the suspension was vigorously hand shaken for 2 min as described by Abia et al. (2015a). The water displacement method, which is important to detach connected microorganisms from the sediment matrix and for the subsequent filtration step onto membranes, does not influence the growth of target bacteria (Abia et al. 2015a). This method was developed in order to better compare microbial counts of sediment and water with one unit (CFU/100 mL) since other studies (Alm et al. 2003; Fries et al. 2008) expressed water and sediment in different units such as MPN GDW or CFU GDW versus CFU/100 mL. One hundred millilitres of aliquots, along with tenfold serial dilutions of the resulting phosphate buffer solution were then analysed using the membrane filtration technique according to standard methods (US EPA 2002a, b). For faecal coliforms, plates were incubated for 24 h at 44.5 °C and for *Enterococcus* spp., plates were incubated for 24–48 h at 37 °C. According to Abia et al. (2015a), results obtained from riverbed sediments by water displacement methods for faecal coliform and *Enterococcus* spp. counts were reported as colony-forming units (CFUs) per 100 mL of sediment suspension in PBS and for river water as CFU/100 mL (Abia et al. 2015a).

### Molecular analysis of faecal coliforms and Enterococcus species

For the molecular analysis of bacterial isolates, five colonies per plate was randomly selected based on colony appearance. Isolated bacteria were preserved in 15% glycerol at 0 °C for molecular analysis.

#### Total genomic DNA extraction of faecal coliforms and *Enterococcus* spp

A total of 53 isolates (14—water and 15—sediment faecal coliforms isolates and 12—water and 12 sediments *Enterococcus* spp. isolates) were used for the molecular study. The preserved bacteria were allowed to thaw, and then centrifuged for 1 min at 12,000 r/min. Total genomic DNA was extracted from the bacterial pellets using the InstaGene™ matrix (Bio-Rad, South Africa) according to the manufacturer’s instructions. The quality and the quantity of the isolated nucleic acids were determined using the NanoDrop™ 2000 spectrophotometer (Thermo scientific) and 1% agarose gel electrophoresis (Bio-Rad, South Africa).

#### PCR amplification of the 16S rRNA gene of *E. coli* and 23S rRNA of *Enterococcus* spp

For the amplification process, the following primer sets were used: 27F (5′-AGAGTTTGATCCTGGCTCAG-3′) and 1507R (5′-CGGGTAACGTCAATGAGCAAA-3′) targeting the 16S rDNA of faecal coliforms (Lane 1991; Heyndrickx et al. 1991); ENT765F (5′-TCTCATCGGCTCCTACCTATC-3′) and ENT1699R (5′-AAGCTGTGGACTACACCATTAG-3′) targeting the 23S rRNA of *Enterococcus* spp. Reactions were run using SsoFast™ EvaGreen® Supermix (Bio-Rad, South Africa) containing 2× reaction buffer with dNTPs, Sso7d-fusion, polymerase, MgCl_2_ and EvaGreen dye and in a resulting volume of 20 µL, consisting of 10 µL of Supermix, 0.5 µL of each primer (concentration 500 µM), 10 ng of isolated gDNA template and 4 µL nuclease-free water (Fermentas, 140 Leon-Rot, Germany). The PCR reactions were carried out in a CFX96™ Real-time PCR Detection System (Bio-Rad, South Africa), and the following thermal cycling conditions were used: enzyme activation step at 98 °C for 2 min, followed by 40 amplification cycles of denaturation at 98 °C for 5 s annealing of primers with the gDNA template at 59 °C for faecal coliforms and 54 °C for *Enterococcus* spp., and a primer extension at 72 °C for 2 min. The specificity of the assay was assessed by the analysis of the melting curve (Fey et al. 2004; Varga and James 2005). Melting curve analysis was performed from 59 °C for faecal coliforms and from 54 °C for *Enterococcus* spp. up to 95 °C with increments of 0.5 °C per 10 s. The melting temperature was defined as the peak of fluorescence in the generated melting curve.

#### Gel electrophoresis

The PCR products were loaded on 1% (w/v) agarose gel for electrophoresis and then stained with ethidium bromide, followed by visualisation under ultraviolet light. The FastRuler Middle Range DNA Ladder (Fermantas) was included in all gels as a size marker. These results were captured using a gel documentation system (Syngene, Cambridge, UK).

#### Sequencing of the 16S rRNA and 23S rRNA genes and sequence analysis

Following the PCR reaction, 29 amplicons for faecal coliforms and 24 amplicons for *Enterococcus* spp. were sequenced using the conventional Sanger (dideoxy) sequencing in the forward direction using the primers described above. Prior to sequencing, the DNA products were purified using PCR clean up kit (Biocombiotech, South Africa) and check for quality using NanoDrop™ 2000 spectrophotometer (Thermo scientific). For sequencing procedure, “BigDye” for ABI3130XL was used according to the manufacturer’s instructions and the gel was run on a 3130XL sequencer. Sequences were analysed by comparing them with known 16S rRNA and 23S rRNA sequences using the BLASTn algorithm (http://blast.ncbi.nlm.nih.gov/Blast.cgi) to find the closest match in GenBank, EMBL, DDBJ, and PDB sequence data. Most similar type species that showed 97% sequence similarity with the isolates were selected as identical species. The 16S rRNA faecal coliform and 23S rRNA enterococci sequences were aligned by Clustal X2 (Larkin et al. 2007) and were then edited using BioEdit v.7.2.5 software (Hall 1999). The distances for each 16S rRNA and 23S rRNA were calculated by the neighbour-joining method (Tamura et al. 2004) and phylogenetic trees were created by using MEGA6 (Tamura et al. 2013). The evolutionary distances for each 16S rRNA and 23S rRNA were calculated by the neighbour-joining method with Kimura 2-parameter model by 500 replicates (Tamura et al. 2004). All locations having gaps and missing data were removed from the data set using the complete-deletion option.

All the newly sequenced bacteria were deposited in the DNA Database of Japan with the accessions number listed in the Additional file 1.

### Statistical analysis

The data were statistically analysed using the IBM SPSS Software (v.22) and Microsoft Excel 2010. The bacterial counts of the river water and riverbed sediment samples were assessed for differences by using a two-way ANOVA with a 95% confidence interval. Spearman’s rank correlation (a non-parametric test of correlation) was used to analyse differences or correlations in the datasets for faecal indicators between water and sediment samples. A Student’s t test was used to investigate any statistically significant differences between the mean values of the microbial loads.

## Results

**Fig. 2:**
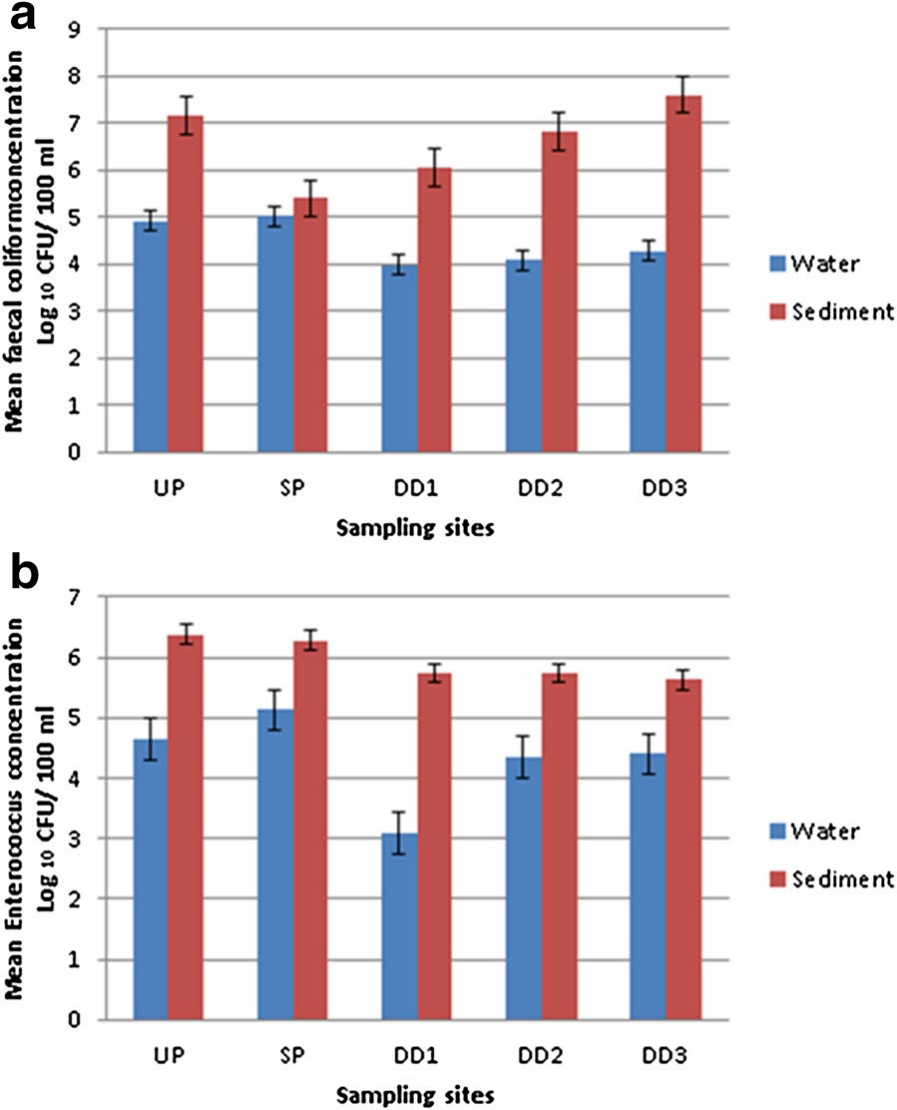
Water vs. sediment concentrations of different sites on the Apies River, South Africa for **a** Faecal coliforms and **b** *Enterococcus* spp

The differences in faecal coliform (Fig. 2a) and enterococci (Fig. 2b) loads between river water and riverbed sediment were observed. Sediment faecal coliform counts were consistently higher than water faecal coliform counts at all sites during the entire sampling period. Faecal coliforms were more abundant than *Enterococcus* with mean concentrations of 7.60 log_10_ and 6.38 log_10_ CFU/100 mL respectively for riverbed sediment and mean concentration of 3.09 log_10_ CFU/100 mL and 3.97 log_10_ CFU/100 mL for river water. Furthermore, the faecal coliform concentrations in the riverbed sediments were significantly higher than in their corresponding overlying water for all sites tested for faecal coliform (UP p < 0.05; SP p < 0.05; DD1 p < 0.05; p < 0.05; DD3 p < 0.05) and Enterococci (UP p < 0.05; SP p < 0.05; DD1 p < 0.05; DD2 p < 0.05; DD3 p < 0.05).

Correlation analysis was used to determine the relationship between river water and riverbed sediment counts at different sampling points along the Apies River. The Spearman’s rank correlation coefficients and the corresponding levels of significance (p values) for the different parameters was also evaluated. For faecal coliforms, 0–0.14 correlation coefficient between river water and riverbed sediment samples were observed in (Table 1).

**Table 1:** Spearman’s correlation predictor variables of faecal coliforms and *Enterococcus* spp

Factors	UP	SP	DD1	DD2	DD3
Faecal coliforms					
r^2^	0.14	0.02	0.05	0.00	0.13
P	<0.05	<0.05	<0.05	<0.05	<0.05
*Enterococcus* spp.					
r^2^	0.65	0.49	0.26	0.45	0.76
P	<0.05	<0.05	<0.05	<0.05	<0.05

### Genetic similarity

The PCR amplification products for the faecal coliforms and enterococci isolated from river water when contrasted with those isolated from riverbed sediments showed a similarity in amplified regions. These results suggest that indicator bacteria isolated from river water might have potential similarity to riverbed sediment isolates. All the samples displayed a single band of 1 500 bp (base pairs) in agarose gel for faecal coliforms and 956 bp for enterococci, indicating the successful amplification of the 16S rRNA and 23S rRNA gene sequence from the isolates.

**Fig. 3:**
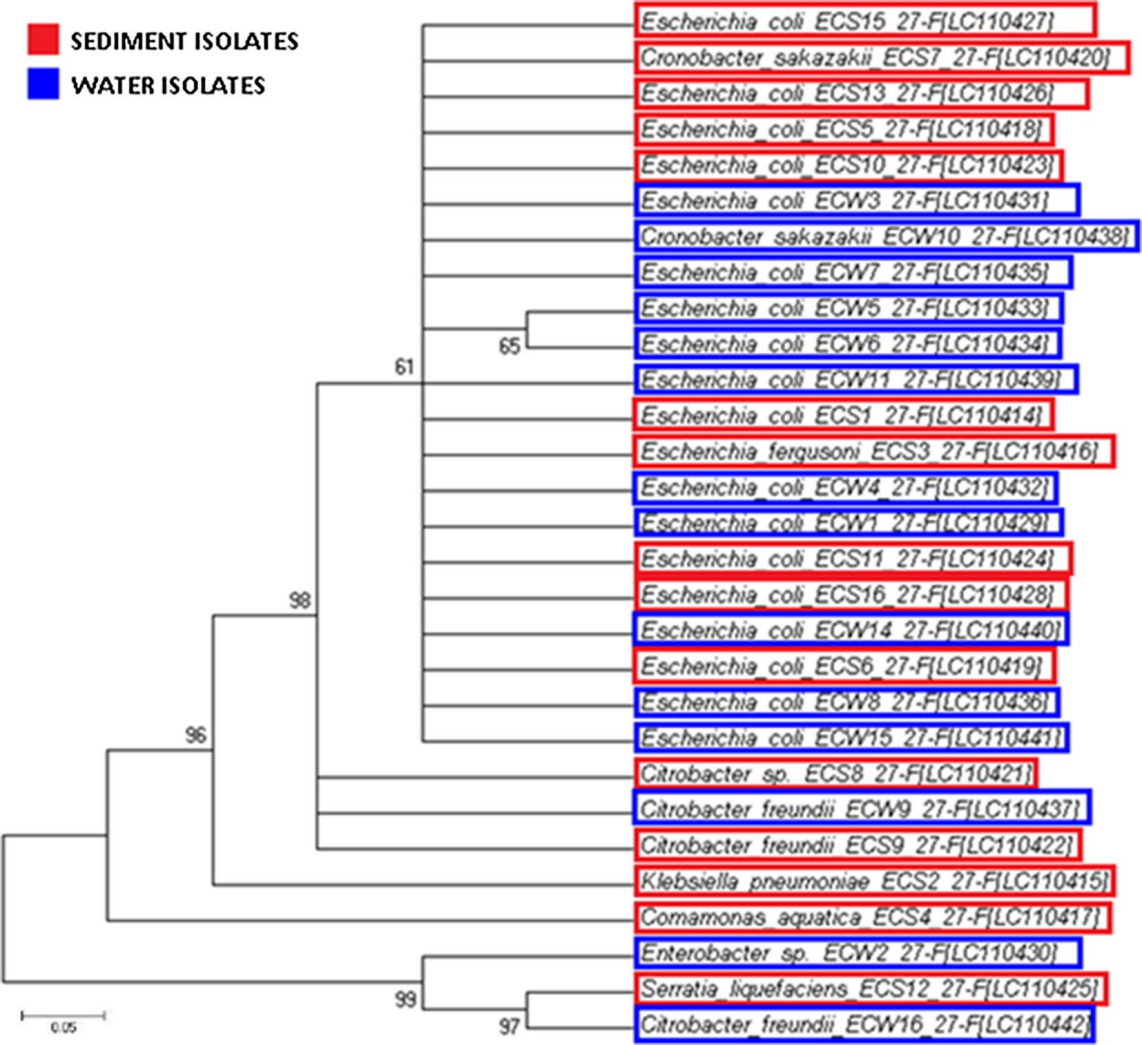
Phylogenetic tree of faecal coliform bacteria isolated from river water (*blue*) and riverbed sediment (*red*) constructed using MEGA 6 with the Neighbor-Joining method-distance Kimura 2, for a 1500 bp fragment of the 16S rRNA coding region of the Faecal coliform bacteria. *Numbers above branches* show bootstrap values expressed as percentages of 100 replications and distribution of the genetic profiles isolated from different sites of the Apies River

**Fig. 4:**
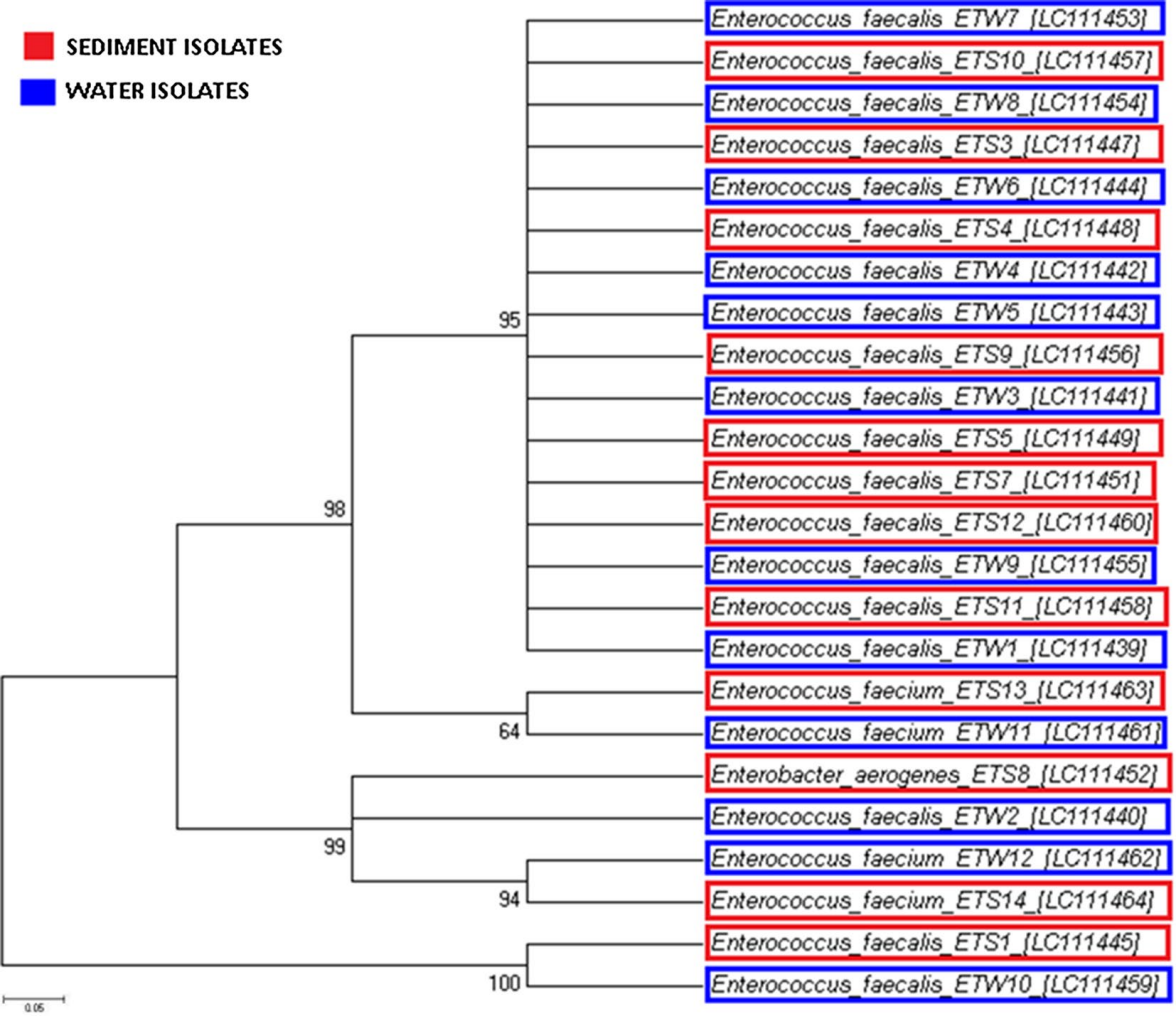
Phylogenetic tree of *Enterococcus* bacteria isolated from river water (*blue*) and riverbed sediment (*red*) constructed using MEGA 6 with the Neighbor-Joining method-distance Kimura 2, for a 934 bp fragment of the 23S rRNA coding region of the *Enterococcus spp*. *Numbers above branches* show bootstrap values expressed as percentages of 100 replications and distribution of the genetic profiles isolated from different sites of the Apies River

The evolutionary history was established utilising the neighbour-joining algorithm (Saitou and Nei 1987) and the optimal tree with the sum of the branch lengths (0.87639720 for faecal coliforms and 2.67726379 for *Enterococcus* spp.) were obtained (Figs. 3, 4). The evolutionary distances were calculated utilising the Kimura-2 parameter method (Kimura 1980) and are in the units of the number of base replacements per location. The analysis involved 29 faecal coliforms and 24 *Enterococcus* spp. nucleotide sequences. First, second and third codon positions were included, while all positions containing crevices and missing information were eliminated from the dataset. A total of 318 (faecal coliform) and 545 (*Enterococcus* spp.) positions were identified in the final dataset. Evolutionary analyses were performed in MEGA 6 (Tamura et al. 2013). All fragments from the phylogenetic tree belonged to the Enterobacteriaceae family for faecal coliforms and to the Enterococcaceae family for enterococci. The sequences recovered from the NCBI nucleotide sequence database that supplied the closest match in pair-wise BLASTn were identified as *E. coli, Citrobacter, Cronobacter, Klebsiella, Serratia, Enterobacter* and *Comamonas* for faecal coliform sequences and *Enterococcus faecalis* and *Enterococcus faecium* for enterococci sequences.

Figure 3 shows the phylogenetic analysis of the faecal coliforms in both the river water and riverbed sediment. *Escherichia coli* (65.52%) was the predominant faecal coliform isolated in the Apies River samples, followed by *Citrobacter* (13.79%), *Cronobacter* (6.89%), *Klebsiella* (3.45%), *Serratia* (3.45%), *Enterobacter* (3.45%), and *Comamonas* (3.45%). The results showed that *E. coli* occurred abundantly in the river water, and clusters of *E. coli* with closely related strains were identified in riverbed sediment samples, while *Klebsiella*; *Serratia* and *Comamonas* were isolated only from the riverbed sediment samples.

## Discussion

Concentration of faecal coliforms and *Enterococcus* spp. in water and sediments of Apies River demonstrates that the river receives high loads of external faecal pollution. This study also revealed significant differences in faecal coliforms and *Enterococcus* spp. loads between the Apies River sediment and water samples. These findings corroborate those of Abia et al. (2015b, c) who reported high *E. coli* levels in water and riverbed sediments of Apies River. The abundance of *E. coli* in Apies River sediments measured by membrane filtration in the present study were comparable to those of Colilert®-18 methods previously reported in riverbed sediments of the Apies River (Abia et al. 2015c). Walk et al. (2007) have also outlined the survival and growth of faecal bacteria in aquatic sediments and also pointed out that sediments may represent their secondary habitat after the intestinal tract of warm-blooded animals which serves as their primary habitat. Sediments can be “reservoirs” of metabolically active faecal indicator bacteria (Pianetti et al. 2004). This has also been confirmed by studies conducted in subtropical areas, where *E. coli* and enterococci displayed higher growth and survival rates in marine sediments than in the overlying seawater (Hartz et al. 2008). When bacteria enter the river, they may form flocs and settle in the bottom of rivers by adhering to sand, rocks, and other particulate matter where they can live and thrive for long periods of time (Craig et al. 2004). This causes the faecal coliforms and enterococci loads in the sediment to be higher than in the overlying water, except during heavy rainfall and runoff events when the load of bacteria in the water increases prior to settling out into the sediment (Orear and Dalman 2011).

Stumpf et al. (2010) examined the input of faecal coliforms during storm flow and base flow, and observed that during storm flow the bacterial load on average was 30–37 times greater than the base flow bacterial loads. Further, the sediment *E. coli* counts reached log_10_ 2.89 CFU/100 mL, well above the EPA proposed safe limit of log_10_ 2.37 CFU/100 mL, and hence sediment acted as a reservoir and source of faecal contamination to the overlying water (Stumpf et al. 2010). Similar results were seen in the Apies River study where sediment faecal coliform counts were as high as log_10_ 7.61 CFU/100 mL. At this concentration and also taking into consideration re-suspension, the findings of this study suggest sediments in Apies River to be a major source of bacterial contamination during periods of low river flow. When sediment and water faecal coliform levels were compared, it was found that on average the bacterial concentration in the sediment was 1- to 3000-folds higher than in the overlying water. Sediments consistently had faecal coliform concentrations that ranged from 1 to 383 times greater than the adjacent water column (Orear and Dalman 2011). In our study, only 21.9% of all the water samples analysed were found to fall within the target water quality range (TWQR) as set out in the *South African Water Quality Guidelines* for *Domestic Water Use* (Volume 1) and for *Recreational Water Use* (Volume 2) (DWAF 1996). In terms of the national standards set by SANS 241 (SABS 2006) and the *South African Water Quality Guidelines* (Volume 1) (DWAF 1996), the limit for faecal coliforms is 0 CFU/100 mL for water that is meant for domestic use; any concentration ≥500 CFU/100 mL in drinking water can lead to gastrointestinal (GI) illness. For recreational activities the minimum acceptable risk is 8.5% GI illness risk in terms of the microbiological indicators given in the *South African Water Quality Guidelines* (Volume 2) (DWAF 1996). The Skinner Spruit (SP) water and sediment close to the Daspoort WWTP recorded the highest levels of both faecal coliform and *Enterococcus* spp. According to the most recent South African Green Drop Progress Report (DWA 2012), this treatment plant with a microbiological compliance level of 87.3% has been treating the wastewater adequately, while the Skinner Spruit, a tributary of the Apies River, is reported to carry elevated microbial loads and thus negatively impacts the water quality of the effluent discharged by this WWTP. High counts were also observed after rainfall events, confirming the findings of Pandey et al. (2012) where a sudden increase in faecal coliform counts is mostly the result of both surface water run-off and re-suspension of the stream bottom sediment.

The enterococci levels found in water were 2–4 logs lower than those found in riverbed sediment. These counts were consistently higher than those of faecal coliforms during the entire sampling period in Fig. 2b. Ferguson et al. (2005) found enterococci to be present in all the samples taken for the study conducted. The geometric mean enterococci concentrations in sediment samples were also higher (log_10_ 3.77 CFU/10 g) than the faecal coliform concentrations (log_10_ 3.27 CFU/10 g). Their occurrence in both water and sediment may suggest a movement between these two environments.

Unlike in other studies, the number of faecal coliforms enumerated from the riverbed sediment samples did not correlate with those enumerated from the river water samples (Craig et al. 2004). A similar attempt to correlate *E. coli* densities in water and in sediment by Valiela et al. (1991), An et al. (2002) and Byappanahalli et al. (2006) was also not successful. This indicated that the faecal coliform densities in water did not relate with the densities in riverbed sediments, leaving the *Enterococcus* spp. to be more related faecal indicators of riverbed sediment. Many studies have proven the *Enterococcus* spp. standard to be the more sensitive compared to faecal coliforms for recreational purposes (Noble et al. 2003; Benedict and Neumann 2004; Neumann et al. 2006).

The faecal coliforms isolated exhibit a 98% nucleotide sequence homology which emphasises the positive correlation between faecal coliforms in river water and riverbed sediment, as also reported earlier (Grant et al. 2001; Boehm et al. 2002; Kim et al. 2004; Noble and Xu 2004). However, these results can also imply a common ancestry and thus likely derived from the same source. Using the phylogenetic tree, Njage and Buys (2015) reported the genetic similarity between commensal and pathogenic *E. coli* strains from lettuce and irrigation water, even at a distance of 246 km apart.

Unlike faecal coliforms, the phylogenetic tree of enterococci, shown in Fig. 4, revealed that *Enterococcus faecalis* was the predominant species found in all the river water and riverbed sediment samples, findings corroborating those of Ferguson et al. (2005). With a bootstrap value of 100%, this may suggest that *Enterococcus* spp. isolated from river water and riverbed sediment samples may be highly similar at the molecular level. Anderson et al. (1997) found that enterococci of marine sediment were suggestive of natural or environmental sources of contamination to overlying water.

Microbial loads of faecal coliforms and enterococci in Apies River revealed a river that is highly polluted with faecal contamination. There is thus a need to rehabilitate or restore this stream to its once pristine state. The correlation coefficient study revealed enterococci counts to be the better indicator than faecal coliforms in search of predicting the degree of contamination in the event of riverbed sediment re-suspension. The present study revealed a high prevalence of faecal indicator bacteria and provided evidence of the close genetic similarity between isolates from river water and riverbed sediment. A 98% homology among the nucleotide sequences between river water and riverbed sediment isolates indicated their close genetic similarity. There is therefore a need to incorporate sediment quality monitoring as an integral component of the nationwide routine surface water quality monitoring programmes. This study recommends future studies to be conducted on a multi-locus sequencing or whole genome sequencing techniques in order to emphasize or reject the finding of this study.

## Additional file

**Additional file 1.** Additional material.

## Abbreviations

16S rRNA: sixteen subunit ribosomal RNA
23S rRNA: twenty-three subunit ribosomal RNA
BLASTn: basic location alignment search tool for nucleotide
CFU GDW: colony-forming units per gram dry weight
CFU GWW: colony-forming units per gram wet weight
CFU: colony forming unit
DDBJ: DNA Data Bank of Japan
DNA: deoxyribose nucleic acid
EMBL: European Molecular Biology Laboratory
gDNA: genomic DNA
MDG: millennium development goals
MEGA6: Molecular Evolutionary Genetics Analysis version 6
MF: membrane filtration
PCR: polymerase chain reaction
MPN GDW: most probable number per gram dry weight
RHP: river health program
TUT: Tshwane University of Technology
UN: united nations
UP: upstream Daspoort Wastewater treatment plant-WWTP
US EPA: United States Environmental Protection Agency
WWPT: wastewater treatment plant
