# Supplementary material for: Genetic relatedness of faecal coliforms and enterococci bacteria isolated from water and sediments of the Apies River, Gauteng, South Africa
Source: AMB Express. 2017 Jan 7;7:20. doi: 10.1186/s13568-016-0319-4 (PMC5218955; doi:10.1186/s13568-016-0319-4)
Supplement: Supplementary file 1 — Additional file 1. Additional material. [file 13568_2016_319_MOESM1_ESM.docx]

Additional material

All the newly sequenced bacteria were deposited in the DNA Data Bank of Japan (DDBJ) with the following accession numbers: LC111465 (SALW1), LC111466 (SALW2), LC111467 (SALW3), LC111468 (SALW13), LC111469 (SALW14), LC111470 (SALW12), LC111471 (SALW11), LC111472 (SALW10), LC111473 (SALW9), LC111474 (SALS12), LC111475 (SALW01), LC111476 (SALW8), LC111477 (SALS1), LC111478 (SALS2), LC111479 (SALW7), LC111480 (SALS11), LC111481 (SALS10), LC111482 (SALW6), LC111483 (SALS9), LC111484 (SALS8), LC111485 (SALS7), LC111486 (SALS6), LC111487 (SALS5), LC111488 (SALS4), LC111489 (SALW5), LC111490 (SALS3) LC111491 (SALW4), LC111492 (SHIGW2), LC111493 (SHIGS1), LC111494 (SHIGW3), LC111495 (SHIGW4), LC111496 (SHIGW1), LC111497 (SHIGS2), LC111498 (SHIGS3), LC111499 (SHIGS4), LC111500 (SHIGW5), LC111501 (SHIGS5), LC111502 (SHIGW6), LC111503 (SHIGW7), LC111504 (SHIGW8), LC111505 (SHIGS6), LC111506 (SHIGS7), LC111507 (SHIGW9), LC111508 (SHIGS8), LC111509 (SHIGW10), LC111510 (SHIGS9), LC111511 (SHIGS10), LC111512 (SHIGS11), LC111513 (SHIGS12), LC111514 (SHIGW11), LC111515 (SHIGS13), LC111516 (SHIGW14), LC111517 (SHIGW13) and LC11151 (SHIGW12)
